# New Insights into Boxer’s Knuckle Injury of the Little Finger

**DOI:** 10.3390/jcm13010046

**Published:** 2023-12-21

**Authors:** Sébastien Durand, Thierry Christen, Jean-Baptiste Ledoux, Romain Baillot

**Affiliations:** 1 Department of Hand Surgery, Lausanne University Hospital, Rue du Bugnon 46, CH-1011 Lausanne, Switzerland; thierry.christen@chuv.ch (T.C.); romain.baillot@chuv.ch (R.B.); 2 Department of Diagnostic and Interventional Radiology, Lausanne University Hospital, University of Lausanne, CH-1011 Lausanne, Switzerland; jean-baptiste.ledoux@chuv.ch

**Keywords:** boxer’s knuckle, extensor digiti minimi, tendon injury, 3D imaging, MRI, ultrasound

## Abstract

Background: The original description of boxer’s knuckle injury of the fifth ray mentions that the injury occurs between the extensor digitorum communis (EDC) and the extensor digiti minimi (EDM). Subsequent reports claim similar findings. Anatomical studies show that the EDC of the fifth ray is absent in most patients, while the EDM is generally composed of two slips. We present a modification of the current description of boxer’s knuckle injury of the little finger based on the correlation between advanced preoperative 3D imaging and intraoperative findings. Methods: Five patients were investigated preoperatively using high-resolution ultrasound and 3D tendon reconstruction-based MR arthrography. Surgical exploration identified the lesion site relative to the EDM and EDC. Results: All patients had two slips of the EDM and no EDC to the fifth ray. The injury appeared as a longitudinal tear of the EDM between its two slips. The mean gap was 7.8 mm (range 4.5–10 mm) on the pathological side vs. 1.3 mm (range 1–2 mm) on the healthy contralateral side. Conclusions: We believe that previous descriptions of boxer’s knuckle of the fifth ray are inaccurate. High-resolution ultrasound and 3D reconstructions based on MR arthrography are reliable diagnostic tools allowing to locate the injury with precision.

## 1. Introduction

The term “boxer’s knuckle” was first used in 1957 [1] to describe post-traumatic pain and swelling on the dorsal aspect of the metacarpophalangeal (MCP) joint in professional boxers. Gladden [1] classified it into four types, according to the extent of injury. This lump corresponds to various tendinous and capsular injuries in zone five. Intra-articular lesions such as synovitis, cartilaginous rice bodies, and chondral infraction of the metacarpal head were also present in several instances [2]. According to some authors [2,3], the injury affects predominantly the middle finger with damage occurring most frequently on the radial sagittal band, which can lead to ulnar subluxation of the extensor tendon. Boxer’s knuckle injury of the little finger is invariably described as a divergent dislocation of the extensor digiti minimi (EDM) and extensor digitorum communis (EDC) tendons. However, illustrations and observations found in clinical reports do not correlate well with the descriptions appearing in anatomical works [3,4,5,6,7,8,9,10,11]. When observing the intraoperative photographs appearing in several of these articles [3,4,7,9], we believe that the two tendinous slips of the EDM were incorrectly identified as the EDM and EDC. Similarly, reports related to the radiological literature mention the same confusion [12]. According to numerous anatomical studies, the EDC of the little finger is absent in the majority of patients [13,14,15,16,17,18,19] and the EDM has two slips in most cases [20]. There are several variations, but the most common pattern includes two slips of the EDM, an absent EDC, and a type 3 juncturae tendinum (JT) in the fourth intermetacarpal space [19] in most of the cases, as described by von Schroeder et al. [20]. Therefore, the probability to observe one EDC and one EDM tendon in all cases of boxer’s knuckle of the little finger is extremely low if not implausible.

The purpose of this retrospective study was to specify the anatomy of boxer’s knuckle injury of the little finger by correlating advanced preoperative imaging and intraoperative findings.

## 2. Materials and Methods

This study was conducted according to the guidelines of the Declaration of Helsinki. We obtained written informed consent for each patient.

Five patients with a boxer’s knuckle injury of the little finger were investigated preoperatively using high-resolution, dynamic ultrasound and 3D reconstructions based on MR arthrography of the fifth MCP joint.

High-frequency ultrasound was performed using an Aixplorer™ system (Supersonic Imagine, Aix-en-Provence, France) with a high-resolution 5–18 MHz linear array transducer. We evaluated the extensor tendons morphology, dynamics and vascularization in B-mode and color Doppler in the transverse/axial and longitudinal planes.

Bilateral MCP MRIs, with arthrography on the pathological side, were obtained on Siemens Magnetom PrismaFit 3T and VidalFit 3T MRI scanners (Siemens healthineers, Erlangen, Germany) using a T1 SPACE (Sampling Perfection with Application optimized Contrasts using different flip angle Evolutions) sequence with an isotropic voxel measuring 0.3 mm^3^. Based on MR images, we reconstructed in three dimensions the following structures with a 3D Slicer (Surgical Planning Laboratory, Brigham and Women’s Hospital, Harvard Medical School, Boston, MA, USA): fifth metacarpal, proximal phalanx of the little finger, EDM, EDC of the little finger if present, and JT between the ring and little fingers.

All patients underwent surgery. Dorsal longitudinal MCP joint incisions were used. The skin flap was carefully elevated, and we identified the site of the lesion relative to the EDM, EDC, and JT, and recorded the type of JT. The scarred area was excised, and the capsule was exposed and assessed for a tear. A torn capsule is repaired only if this can be performed without tension in 90° of MCP flexion. A running suture with 4–0 nonabsorbable Ethibond suture (Ethicon Inc., Somerville, NJ, USA) was used to close the gap and repair the extensor tendon. The fifth ray MCP joint was immobilized in extension for three weeks. Gentle active flexion and extension of all finger joints was started after 3 weeks. Range of motion of the MCP joint, swelling, grip strength (Jamar dynamometer), pain, and Quick DASH were recorded after a minimum of 6 months follow-up.

## 3. Results

**Figure 1:**
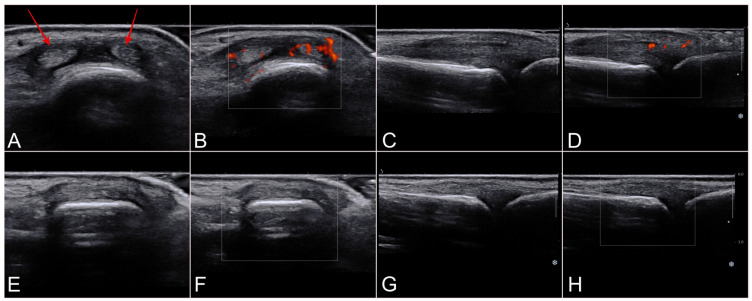
Preoperative high-resolution ultrasound (case 4, 24-year-old female). Transverse/axial ages in B-mode (**A**), color Doppler (**B**), sagittal images in B-mode (**C**) and color Doppler (**D**) showing hyperemia and (red arrows) gap through the EDM on the pathological side. Contralateral comparative images (**E**–**H**).

Table 1 shows patients’ characteristics and clinical outcomes. Four males and one female with a mean age of 36 years (range 25–49 years) complained of a painful lump on the dorsal aspect of the MCP joint of the little finger with painful MCP extension. One patient had a concomitant fracture of the fifth metacarpal. High-resolution ultrasound showed swelling with hyperemia of the extensor mechanism on the dorsal aspect of the MCP joint. The injury appeared as a longitudinal tear with a gap between two tendons (Figure 1). The gap increased with MCP joint flexion.

**Figure 2:**
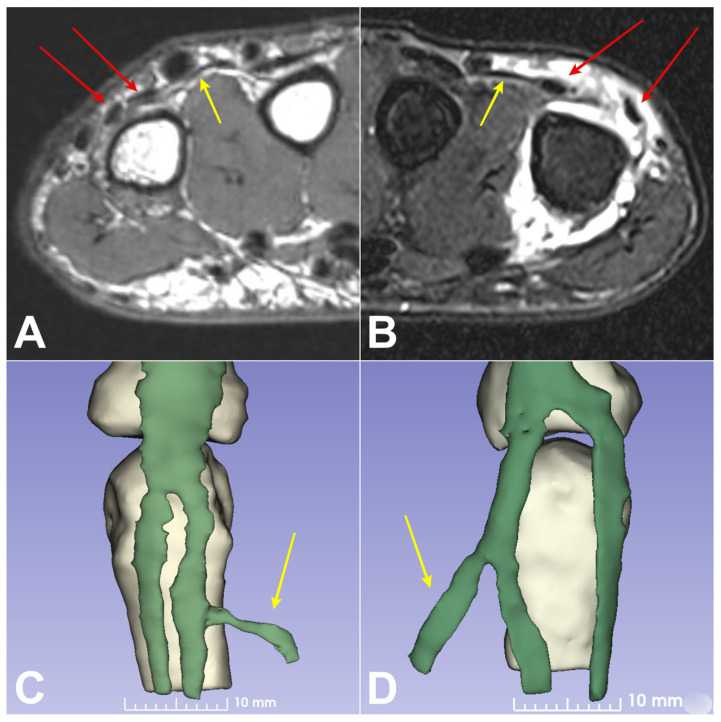
Representative MR images (case 1, 25-year-old male). Axial T1-weighted on the healthy side (**A**) and axial fat-suppressed T2-weighted with arthrography on the pathological side (**B**). MRI revealed a gap between the two tendon slips of the EDM (red arrows) on the pathological side. Using 3D reconstruction based on MRI, the maximum distance between the two tendons of the EDM was 7.2 mm on the injured side (**D**) and 1.5 mm on the contralateral side (**C**). The JT of the fourth inter metacarpal space (yellow arrow) was in continuity with the EDM on the pathological and contralateral side.

**Figure 3:**
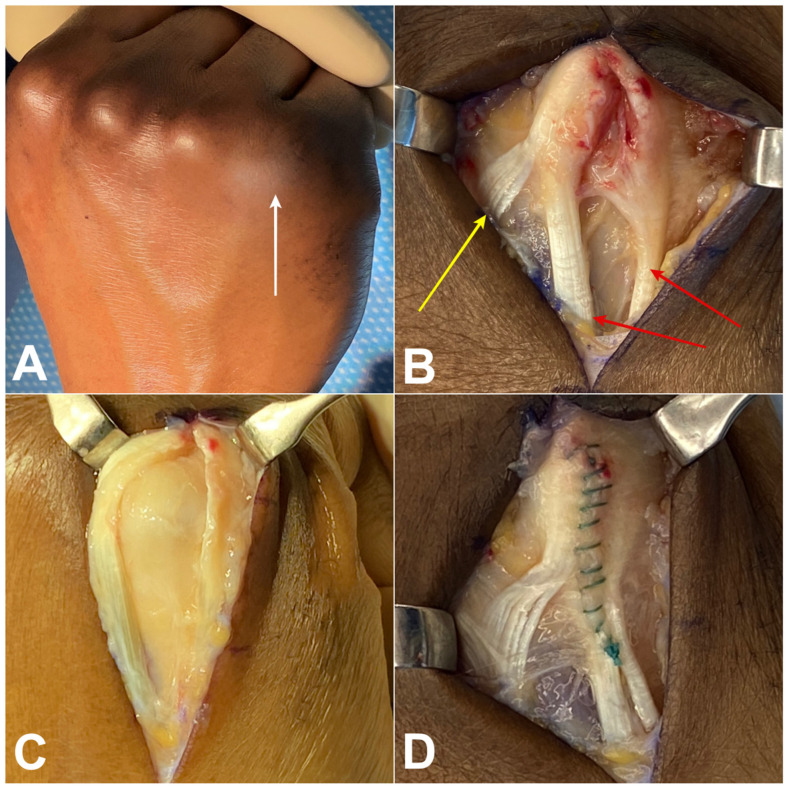
Preoperative photograph (case 3, 27 year-old male) of the dorsal aspect of the hand showing swelling (white arrow) over the 5th MCP joint (**A**). Intraoperative photographs showing the gap between the two tendons (red arrows) of the EDM (**B**) that increases with MCP flexion (**C**) Results after running suture of the EDM to close the space between the two tendinous slips (**D**). Note that JT type 3y (yellow arrow) remains attached to the radial tendon of the EDM.

Bilateral MCP joint MRI with arthrography on the pathological side confirmed a similar longitudinal tear with a gap (Figure 2). The two tendons were traced proximally; both went through the fifth extensor compartment, confirming they were both slips of the extensor digiti minimi. A JT joined the slip tendon of the EDM from the EDC of the ring finger in all patients (Figure 2). The 3D reconstruction of the extensor tendons confirmed a gap with subluxation of the two EDM tendons on the dorsal aspect of the MCP joint in comparison to the healthy contralateral side (Figure 2C,D). The mean gap between the two tendons was 7.8 mm (range 4.5–10 mm) on the pathological side and 1.3 mm (range 1–2 mm) on the contralateral side. Surgical exploration visualized the tear between the two tendons of the EDM in all cases. The EDC of the little finger was absent in all cases, and JT between the EDC of the ring finger and the radial tendon of the EDM were observed in all patients (Figure 3). At a median follow-up of 16.8 months (range 6–60 months) all patients were pain free at rest with an excellent Quick DASH score. No complications were seen. Preservation of grip strength and complete range of motion of the MCP joint of the little finger were observed for all patients. 

## 4. Discussion

In a meta-analysis with a pooled sample of 2247 hands [21], the EDM was present in nearly 100% of cases. The most frequent configurations were two slips in 77.6%, one slip in 11.5%, and three or four slips in, respectively, 7% and 0.6%. In 12% of cases, an accessory slip of the EDM was identified going towards the ring finger. The EDC to the small finger is absent in the majority of the hands [14,17]. Reports show a wide range (9% to 100%) of presence of the EDC to the small finger, probably because the JT from the EDC of the ring finger to the small finger may be mistaken for the EDC of the small finger [18]. Three distinct types of JT have been described. Type 1 juncturae are thin and filamentous and are found primarily between EDC tendons to the index and middle fingers and between the tendon to the middle and ring fingers. Type 2 juncturae are thicker and well defined and are present between extensor tendons to the middle and ring fingers and between the tendons to the ring and little finger, and type 3 juncturae consists of a tendinous slip between EDC of the ring finger and the EDM. Two subtypes of the type 3 juncturae are identified based on their appearance. A type 3y represents a tendon that splits to insert into two adjacent fingers A type 3r is a more transverse junctural connection between tendons [20]. The JT of the fourth intermetacarpal space is present in 92% of the cases with a type 3 in 84% [14]. We observed one type 2 and four type 3 JT (two type 3r and two type 3y) in our patients. A longitudinal tear of the EDM in boxer’s knuckle of the little finger, which has not been described yet, can be explained by the presence of two tendinous slips in all of our cases. The ulnar and radial tendons are located on either side of the central longitudinal axis of the MCP joint [22]. These two tendons are bound together by a thin intertendinous fascia, which is a weak point when a direct blow occurs centrally on the head of the fifth metacarpal. One of our cases, which had a combined fracture of the fifth metacarpal and longitudinal tear of the EDM, very likely had the same mechanism of injury. Closed EDM rupture associated with fifth metacarpal fracture has been reported previously [23].

In boxer’s knuckle, conventional radiography showed no fracture. Abnormal signal intensity or echogenicity of the thickened and possible discontinuity of the sagittal band with edema of the dorsal soft tissues suggested the diagnosis on MRI and ultrasound. Dynamic ultrasound can confirm the extensor tendon subluxation during MCP joint flexion, which is an advantage compared to static MRI. While dynamic ultrasound allowed us to diagnose the damage to the extensor mechanism and instability of the extensor tendons over the MCP joint [24], it is actually the MRI—as suggested by other authors [3]—that confirmed the diagnosis with a high certainty in every case, and identified capsular and intraarticular lesions. The 3D reconstruction based on MRI images made it possible to distinguish the EDM and JT from the tendon of the EDC. In clinical practice, 3D bone reconstruction is typically obtained from CT images since it is more difficult to carry out from MRI images, especially for soft tissues. Currently, various segmentation techniques (threshold-based, region-based, edge-based, clustering-based, artificial neural network-based, atlas-based) are being developed and will improve our ability to carry out 3D reconstruction from MRI images [25]. In our cases, the segmentation was performed manually. While this means that it could be performed by any practitioner, it requires a substantial time commitment for 3D reconstruction with an output that may be slightly deformed.

It may appear unlikely that the description of boxer’s knuckle injury has been erroneous for more than thirty years. However, our clinical findings are in accordance with multiple anatomical studies of the extensor tendons. While the treatment remains identical whether the tendinous tear occurs between two slips of the EDM or between the EDC and the EDM, surgeons should be aware of the anatomy of the extensor mechanism of the little finger to avoid confusing the EDC for the EDM. Treatment options for boxer’s knuckle are not well established. Older studies [1,2] reported that the duration of symptoms could be from 2 to 5 years. Gladden stated that only one of four patients responded to conservative treatment, while other authors have reported it to be ineffective in all cases [3]. Some authors contend that a joint capsule tear is a strong indication for surgical treatment [3]. However, the surgical intervention at the joint capsule is not routine and must be determined on a case-by-case basis to prevent joint stiffness.

A longitudinal tear has also been reported in the extensor pollicis brevis and peroneus brevis (PB) tendons [26,27]. While the cause of these tears is not known, it is likely traumatic, or the result of the tendon splaying over the edge of bone during tendon subluxation. Split tears of the PB tendon respond poorly to nonsurgical treatment, leading some authors to recommend surgical repair. Surgical management allowed us to do an accurate assessment of the injuries present and to carry out tendon repair with running suture, leading to excellent clinical outcomes.

## Figures and Tables

**Table 1:** Patient characteristics and clinical outcomes.

Case	Age (Years)	Sex	Side	Injury Mechanism	Time before Surgery	EDM/ EDC V/ JT Type (1, 2 or 3)	Inter-tendon Gap * (mm)	Follow-Up Time	Grip Strength * (kg)	MCP Joint ROM (Degrees)	Pain at Rest	QuickDASH Score
1	25	Male	Right	Crush injury (piano)	4 weeks	2 0 2	7.2 (1.5)	5 years	40 (40)	0/90	0	0
2	37	Male	Right	Skateboard fall M5 fracture	10 weeks	2 0 3r	10 (1)	6 months	40 (45)	−10/90	0	2.27
3	27	Male	Right	Punch	2 weeks	2 0 3y	8.9 (2)	6 months	50 (60)	−20/100	0	4.55
4	42	Female	Right	Crush injury	7 weeks	2 0 3r	4.5 (1)	6 months	30 (30)	−10/90	0	0
5	49	Male	Left	Punch	5 months	2 0 3y	8.2 (1)	6 months	60 (60)	−20/100	0	2.27

EDM and EDC V column indicate the number of tendons slips for both. M5 = 5th metacarpal bone; MCP = metacarpophalangeal; ROM = range of motion; EDM = extensor digiti minimi; EDC V: extensor digitorum communis of the little finger; JT: juncturae tendinum. * = values for the healthy contralateral side are indicated in parentheses.

## Data Availability

All data generated or analyzed during this study are included in this article. Further enquiries can be directed to the corresponding author.
